# The impact of a short-term cohousing initiative among schizophrenia patients, high school students, and their social context: A qualitative case study

**DOI:** 10.1371/journal.pone.0190895

**Published:** 2018-01-11

**Authors:** Domingo Palacios-Ceña, Emilio Andrés Martín-Tejedor, Ana Elías-Elispuru, Amaia Garate-Samaniego, Jorge Pérez-Corrales, Elena García-García

**Affiliations:** 1 Department of Physical Therapy, Occupational Therapy, Rehabilitation and Physical Medicine, Rey Juan Carlos University, Alcorcón, Madrid, Spain; 2 Hospital Psiquiátrico San Juan de Dios Arrasate-Mondragón, Hermanos de San Juan de Dios, Arrasate, Spain; 3 Department of Research, Fundación San Juan de Dios, Madrid, Spain; IRCCS E. Medea, ITALY

## Abstract

**Background:**

A number of programs have been developed to promote the contact between adolescents and mentally-ill patients, in order to break the stigma, improve understanding, promote mental health and prevent substance abuse. The aim of this study was to describe the experience of patients with schizophrenia, high school students, and their social context, participating in a short-term cohousing initiative.

**Methods:**

A qualitative case-study approach was implemented. Patients with schizophrenia from the San Juan de Dios Psychiatric Hospital, female students from Almen High School, and participants from their social context (parents, hospital staff, and teachers) were included, using purposeful sampling. Data were collected from 51 participants (15 patients, nine students, 11 hospital staff, six teachers, 10 parents) via non-participant observation, focus groups, informal interviews, researchers’ field notes and patients’ personal diaries and letters. A thematic analysis was performed.

**Results:**

The themes identified included a) learning to live together: students and patients participate and learn together; b) the perception of the illness and the mentally-ill: the barrier between health and disease is very slim, and society tends to avoid contact with those who are ill; c) change: a transformation takes place in students, in their self-perception, based on the real and intense nature of the experience; d) a trial and an opportunity: patients test their ability to live outside the hospital; e) discharge and readmission: discharge is experienced as both a liberation and a difficulty, whereas relapse and readmission are experienced as failures.

**Conclusions:**

Our findings can help us to better understand schizophrenia and encourage a more positive approach towards both the illness and those who suffer from it. These results may be used for the development of cohousing programs in controlled environments.

## Introduction

Schizophrenia is a psychiatric disorder characterized by delusions and hallucinations (positive symptoms), accompanied by impaired motivation, social withdrawal (negative symptoms), and cognitive impairment [1,2]. The positive symptoms tend to relapse, whereas the negative and more cognitive symptoms may have long-term effects on social function. The first episode of psychosis is usually detected during late adolescence or early adulthood [1].

Schizophrenia occurs worldwide, with a reported incidence of roughly 15 men and 10 women per 100.000 population per year, and a point prevalence of 4.6 per 1000 individuals [3]. Genetic factors contribute to the etiology of schizophrenia [1,2,4]. Also, the environmental risk factors for schizophrenia include: neurodevelopmental problems during pregnancy, head injuries, epilepsy, autoimmune diseases, infections, and socioeconomic factors [1,2]. In addition, substance use during adolescence increases the risk of suffering schizophrenia [5–7]. Other potential psychosocial risks include experiencing traumatic situations as a child (such as sexual, physical and/or emotional abuse, or situations of abandonment), and migration [8].

However, according to current psychiatric and medical views, schizophrenia cannot be explained merely by biology or the environment [9]. Other important aspects that may help improve our understanding of the illness and its impact on people, include cultural aspects, beliefs, values, and the social context [10,11]. Although pharmacological treatments for schizophrenia can relieve psychotic symptoms, generally, these drugs fail to provide substantial improvements in social, cognitive and occupational functioning [4]. Several interventions exist to reduce the risk of schizophrenia, including training of social skills, early interventions to prevent drug abuse, and measures to improve resilience [12–15]. Also, psychosocial interventions, such as cognitive-behavioral therapy, cognitive remediation and supported education and employment have additional treatment value, despite being inconsistently applied [4]. Prior studies report a relapse rate of between 57.3%-80% [4,16], and social factors have been found to contribute to 39% of admissions in patients with schizophrenia, followed by factors related to mental and physical disorders (31%) [17]. Additionally, relapses can be devastating for the individual and are associated with a deteriorating course of illness, such as increased levels of psychotic symptoms remaining after each acute episode (residual symptoms) [18]. A number of rehabilitation interventions have been developed to improve functional outcomes and promote recovery [19]. On the one hand, traditional practice using psychopharmacologic, somatic, psychosocial, and system-based interventions, with a central focus on decreasing symptoms and disability [10]. On the other hand, the recovery model focuses on the promotion of the individual’s wellbeing, independence, and the subjective experience of personalized experiential recovery [10]. Recovery-oriented practice includes: a) peer-led mentorship interventions; b) self-management and self-care interventions; c) securing personal and environmental resources for optimal interdependent community living; d) supported employment; and e) supported housing [10]. Moreover, cohousing experiences constitute another type of recovery-oriented practice. These consist of direct exchanges between psychiatric patients and adolescents, and may represent a therapeutic alternative for promoting mental health, and reducing discrimination, helping individuals with schizophrenia in their decision making. Housing is considered a pre-requisite for social integration, owing to the fact that people with schizophrenia are less likely to rent their own apartments [20], and are at a high risk for homelessness [10, 21]. These placements contribute to a decrease in homelessness, the number of hospitalizations, and psychiatric symptoms. Also, supported housing programs have a positive impact on psychosocial outcomes, and the overall quality of life [10].

Individuals with mental illness may be subjected to prejudice and discrimination from others and, as a result, may internalize feelings of devaluation [22–25]. Previous studies [26–28] have reported that the public opinion on mental illness has changed over the last decade, revealing a decrease in articles promoting negative ideas, such as the danger posed by mental illness, as well as an increase in the proportion of anti-stigmatizing articles. Other authors [12,29] have underlined the importance of developing interventions aimed at reducing internalized and public stigma, and the exclusion of those who suffer the loss of their social roles and networks. Furthermore, several initiatives have taken place [29–32] with high school students and students’ family members to increase their knowledge on mental illnesses, decrease the associated stigma, and facilitate the integration of patients, which includes increasing personal contact with people with schizophrenia [29,30].

Several international institutions [33,34] recommend actions for preventing mental illness, and promoting mental health, especially in adolescents, by applying strategies against discrimination and stigmatization and promoting opportunities for people with mental illness. In Spain, these actions include measures to prevent addiction to substances (especially cannabis) among adolescents, as well as involving both the social environment (schools, high schools, work environment) and the family environment within the line of action [35,36]. In Spain, substance use during adolescence increases the risk of suffering schizophrenia and other psychiatric problems [35,36]. By visiting a psychiatric hospital, those who belong to risk groups, such as adolescents living in the community, are given the opportunity to interact and share experiences with hospitalized young patients with a history or ties to substance abuse. Currently, in Spain, the San Juan de Dios Hospital [http://sanjuandedios-mondragon.com/], via its program “Meet the Hospital” (S1 File) promotes the first contact between the issue of mental health and high school students, by raising awareness regarding the abuse of substances, such as cannabis. This program was established in collaboration with the San Juan de Dios Psychiatric Hospital and Almen High School, and includes an informative meeting on mental disorders and schizophrenia, visits to a Psychiatry Hospital, sessions with patients, the study of the evolution and impact of schizophrenia and substance use on the lives of the patients involved, and the meaning of schizophrenia for students, teachers, and the students’ parents.

To date, no studies have described the experience of a cohousing initiative between high school students and mentally ill individuals in contexts beyond the hospital. To the best of our knowledge, no prior studies have described the experiences of patients and youth living together in the same house, in the short-to-mid term. The aim of this study was to describe the experience of a short-term cohousing initiative for a group of patients with schizophrenia, and high school students, and to study the impact of the same on the close social and family context.

## Materials and methods

Qualitative methods are useful for understanding the beliefs, values, and motivations that underlie individual health behaviors [37,38]. Furthermore, qualitative studies have been used to research the experience of living with schizophrenia [39,40], as well as issues regarding stigma [41], relapses [42], and the acquirement of healthy behaviors among individuals with schizophrenia [43].

### Study design

A qualitative descriptive case study with embedded units was conducted [37,44,45,46]. A case study may be formed of different units, which help to describe a phenomenon. These units may be different participants, from different contexts and places who are only connected by the phenomenon under study [44,45,47]. In this study, the phenomenon under study is the impact of a short-term cohousing initiative among different participants including schizophrenia patients and high school students, and other participants in their social and family context, such as hospital professionals, students’ parents, and high school teachers.

### Context

The short-term cohousing experience took place at a guest house (Figure A to G in S1 Photo, S2 File) located in the countryside of Respaldiza (Arrasate/Mondragon, Spain), between September 8–11, 2015, involving 15 patients with schizophrenia from the San Juan de Dios Psychiatric Hospital (SJPH), and nine students from Almen High-School (AH). This cohousing experience was part of the “Meet the Hospital” program of SJPH (S1 File). The main goal of this program is to increase awareness regarding substance abuse and mental illnesses, and promote contact between people with mental illness and the community.

Upon commencement of this study, patients and students included in this cohousing experience were placed in groups. The groups were assigned randomly, attempting to establish the same homogenous number of participants in each group and to encourage contact between people with mental health and the community. Each group was formed by three students and five patients. During the cohousing experience, each of the groups had a series of assigned responsibilities, besides different group activities, where they had to interact and work together (S3 File).

### Participants

We included participants who constituted units of analysis and who could provide information regarding the phenomenon under study (S1 Fig). As the cohousing experience was set within the “Meet the Hospital” program, we included all those who participated in the program and who fulfilled the inclusion criteria.

Inclusion criteria.

a Participants in the cohousing experience: patients and students.
  Patients diagnosed with schizophrenia by their psychiatrist, classified according to the ICD-10 (F 20.0–22, F24-25, F28-31, F32.3 and F33.3), with a severe mental disorder [48], participating in a “Meet the Hospital” program at the SJPH, who were part of an open institutionalization regime at the SJPH during the study (the patient temporarily resides at the hospital with the option of participating freely in the community and sleeping at the complex), with an established illness (absence of positive symptoms or presence of attenuated non psychotic forms, absence of disruptive behavior), without severe intellectual and cognitive decline (Wechsler Scale, WAIS III >70), patients who had demonstrated adherence to the prescribed treatment during the previous three months (confirmed by the follow-up by health professionals from the SJPH), no consumption of drugs or other substances in the week prior to the cohousing (determined by blood test), and individuals with the legal autonomy to sign the informed consent.
  Students from the AH, in their first year of high school baccalaureate (aged between 16–17 years), participating in a “Meet the Hospital” program at the SJPH, signed the informed consent and were granted permission to participate by their parents.
b Involvement of participants from the patients’ context.
  The social context of the patients included professionals of the SJPH, who were involved in attending the health needs of patients participating in a “Meet the Hospital” program at the SJPH, and who signed the informed consent.
c Involvement of participants from the students’ context
  The students’ social and family context was comprised of: students’ parents who were participating in a “Meet the Hospital” program at the SJPH, and who signed the informed consent, and student’s teachers who were participating in a “Meet the Hospital” program at the SJPH, and who signed the informed consent.

The patient exclusion criteria were: a) the presence of cognitive disorders, b) difficulties in comprehension and communication, c) the presence of mental crises and relapses, and d) refusal to participate in the study.

### Sampling strategies

A purposeful sampling strategy was employed [46,49], which involved deliberately selecting participants. In this case: people who participated in the cohousing initiative, and people living in the participants’ context who were participating in a “Meet the Hospital” program at the SJPH [37,49].

We contacted 18 high schools which, at the time of the study, were participating in the “Meet the Hospital” program of the SJPH. Of these, only Almen High school, agreed to participate in this study. In total, 10 teachers and 64 students from the first year of high school baccalaureate studies (aged between 16 and 17 years old) and who had participated in the SJPH program were offered the opportunity to participate in this study. Ultimately, 9 students and 6 teachers agreed to participate. Once the students were incorporated into the study, the parents were contacted and invited to participate. As a result, 11 family members of students were recruited. At the hospital, contact was made with 21 patients who were participating in the “Meet the Hospital” program. Of these, 15 agreed to participate. Once the patients in the study were identified, 17 hospital professionals who had a care relationship with the patients were offered to participate, of whom 11 eventually accepted. Finally, 51 participants were included within the sample and none withdrew from the study (Table 1).

**Table 1:** Sociodemographic data of participants.

Participants	Sociodemographic data
Patients	Participants:15 (4 women) Mean age: 34.46 (SD +/- 8.14) Time of stay (months): 28.33 (SD +/- 13.28)
Students	Participants: 9 women Mean age: 17.88 (SD +/- 0.33)
Hospital professionals	Participants: 11 (8 women) Mean age: 39.27 (SD +/- 5.46) Years of experience in mental health: 9.54 (SD +/- 4.18)
Parents of students	Participants: 10 (5 women) Mean age: 50.1 (SD +/- 2.46)
High school teachers	Participants: 6 (5 women) Mean age: 56.16 (SD +/- 4.44) Years of teaching experience: 32.16 (SD +/- 5.19)

SD: Standard deviation

### Recruitment procedure

The researchers approached a) the medical director of the SJPH, who acted as an intermediary to facilitate contact with patients and professionals and b) the director of the AH, and the director of studies, to enable contact with the students, the students’ parents and the high school teachers. Subsequently, the researchers explained the purpose and design of the study to the patients during an initial face-to-face contact session. Separate informative sessions were held for the patients, their legal tutors, the health professionals, students, teachers and parents from the AH. Thereafter, another group information session was performed with students, their parents, and high school teachers. During these meetings, the study was described and questions were answered. Finally, the participants were invited to participate in the study. These informative sessions took place at the hospital and at the HA.

### Researchers’ backgrounds

The research team consisted of six members (three women and three men), three from SJPH (EAMT, AEE, AGS), one from the Hospitaller Order of Saint John of God Foundation (EGG), and two from Rey Juan Carlos University (DPC, JPC). Two members were psychologists, one was a physiotherapist, two were occupational therapists, and one was a nurse. Four members of the team had clinical experience in mental disorders and psychiatry (JPC, EAMT, AEE, AGS), psychogeriatric (DPC), and rehabilitation (EGG). The remaining authors had no previous contact with any of the participants.

### Data collection

Data were acquired over a period spanning seven months to one year, from September 2015 until March 2016. The objective of this case study was to obtain an in-depth multi-perspective holistic enquiry regarding the phenomena of interest, entailing the need for multiple data sources and multiple data collection tools (Table 2) [47].

**Table 2:** Data collection process.

Data collection tool	Participants	Setting	Time	Study phase
Cohousing participants				
Non-participant observation	15 Patients+ 9 students	Respaldiza House	42 hours (20520 minutes)	During cohousing
7 Informal interview	5 patients+ 2 students.	Respaldiza House	184 minutes	During cohousing
Patients’ context				
Diary	2 Patients	Hospital		Post-cohousing
1 Focus group	11 Patients	Hospital	127 minutes	Post-cohousing
1 Focus group	11 Hospital staff	Hospital	92 minutes	Post-cohousing
Students’ context				
Personal Letter	1 student			Post-cohousing
Diary	3 students			Post-cohousing
1 Focus group	9 High school students	High school	118 minutes	Post-cohousing
1 Focus group	10 Parents of students	Hospital	153 minutes	Post-cohousing
1 Focus group	6 High school teachers	High school	75 minutes	Post-cohousing

#### Non-participant observation

Observational data collection is based on the systematic, detailed observation of people and events in order to explore behaviors and interactions within the participants’ own context [38]. In this study, non-participant observation was conducted during the cohousing experience, where the researcher had no other relationship with the group under observation [46,50]. Also, the researcher collected detailed observational field notes systematically and unobtrusively. Observations were undertaken during: a) programmed activities of work in groups; b) leisure time; c) at breakfast, lunch and dinner; and d) during recreational activities which were programmed outside of the guest house (see S3 File).

During these observation periods, researchers used their field notes to record numerous details, such as: the physical layout of the place, the people involved, the activities that took place, the actions and activities performed, the sequencing of events, and the emotions expressed [50]. During such observation periods, researchers also conducted informal or conversational interviews, which allowed them to discuss and delve further into relevant issues, or ask questions about events [50,51]. Also, the interview usually took place within the context of fieldwork, based on participant observation [46,51]. In this case, the recruitment of patients for the informal conversations took place during the spare time between the scheduled activities, when the patients met to smoke and comment on the activities. The interviewer approached the patients and asked them about the possibility of questioning them regarding their perspectives on the co-housing initiative, performing an informal interview to whoever agreed to participate (only 5 patients). The reason patients were chosen was because these informal interviews enabled the possibility of exploring the perspective of patients first-hand, individually, and accessing information on the co-housing experience that was more personal [46,51]. This information was therefore different to the information gathered using other data collection tools (observation and focus groups) [46].

#### Focus groups

Focus groups (FGs) were conducted to examine different perspectives within the same group, acquire understanding of the problems faced by the group and facilitate the identification of values and norms [46,52]. In total, there were 5 FGs (See Table 2), and each FG comprised between 6–12 participants [46].

The FGs were conducted by a moderator following a uniform structure [46,52]. The moderator posed questions, to which each participant responded, respecting their turn to speak. Subsequently, the moderator posed further questions, based on the issues raised in the discussion, in order to further explore or clarify aspects, either on an individual level or with the group as a whole. Two question guides were used: one for patients and one for the remaining participants [46], these were sufficiently focused to gather information on the area of study, but open enough to stimulate discussion and interaction between the participants [52] (S4–S7 Files).

In the patient FG, researchers took into consideration the listening, processing and conversational skills of the individuals with schizophrenia. The researchers asked concise questions, and rephrased or repeated these as necessary [51]. Also, questions evolved from the general to the particular, i.e. starting with open-ended questions on broad issues, and subsequently focusing on more specific questions [51].

Focus groups were conducted in Spanish, and were audio recorded. Permission for these recordings was sought before the recordings began.

#### Written documents

We also collected personal letters and diaries provided by the patients and students, and the researchers’ field notes. The participants’ personal letters and diaries provided a rich source of information as they described personal experiences from the participants’ point of view [46] (See Table 2).

### Data analysis

A thematic, inductive analysis was performed [46,53]. This type of analysis is congruent with the design by covering the multiple perspectives of the case study participants [47].

Complete and literal transcriptions were drafted for each of the FGs, informal interviews, researchers’ field notes, and for the participants’ letters [46]. Thematic analysis [53,54] consisted of identifying the most descriptive content in order to obtain meaningful units, and subsequently reduce and identify the most common meaningful groups. In this manner, groups of meaningful units were formed, i.e. similar points or content that allowed the emergence of the topics that described the study participants’ experience [46]. This thematic analysis process was performed separately upon the non-participant observations, informal interviews, FGs, diaries and personal letters. Subsequently, joint meetings were held to combine the results of the analysis. Also, the data collection and analysis procedures were discussed during these meetings. In the case of differences in opinion, theme identification was performed based on consensus among the research team members. Subsequently, the research team held joint meetings to show, combine, integrate and identify final themes [47]. No data analysis software was used.

### Quality criteria

The guidelines for conducting qualitative studies established by the consolidated criteria for reporting qualitative research [55] (http://www.equator-network.org/) and the recommendations for the design of Case Study Research in health care using the DESCARTE model [47] were followed. Also, the criteria for guaranteeing trustworthiness as cited by Guba & Lincoln were followed [56]. The techniques performed and the application procedures used to control trustworthiness are described in Table 3. These methods to increase rigor are compatible with case-study designs [45,57].

**Table 3:** Trustworthiness criteria applied.

Criteria	Techniques performed and application procedures
Credibility	Investigator triangulation: each data source was analyzed. Thereafter, team meetings were performed during which the analyses were compared and themes were identified. Triangulation of methods of data collection: including non-participant observation, focus groups, informal interviews, personal letters, diaries and researcher field notes. Participant validation: this consisted of asking the participants to confirm the data obtained at the stages of data collection.
Transferability	In-depth descriptions of the study performed, providing details of the characteristics of researchers, participants, contexts, sampling strategies, and the data collection and analysis procedures.
Dependability	Audit by an external researcher: an external researcher assessed the study research protocol, focusing on aspects concerning the methods applied and the study design.
Confirmability	Investigator triangulation, data collection triangulation. Researcher reflexivity was encouraged via the previous positioning, performance of reflexive reports and by describing the rationale behind the study.

### Ethics

This study was approved by the Clinical Research Ethics Committee of the University Rey Juan Carlos (project number: 051020154215) and the “Hermanos de San Juan de Dios Foundation” (project number: 7-2-12). Permission was also obtained from the SJPH (S8–S10 Files).

The study was conducted in accordance with the principles articulated in the WMA Declaration of Helsinki (Ethical Principles for Medical Research Involving Human Subjects) [58]. Furthermore, we followed the Spanish Personal Data Protection Act [59] and the Biomedical Research Act [60].

Moreover, we considered special ethical considerations for the students and patients with schizophrenia (S11 File).

## Results

Fifty-one participants were included in the study. See Table 1 for the sociodemographic profile of the participants. Fifteen patients (11 male) were included with a mean age of 34.46, and 28.33 months of mean hospital stay. Nine female students participated, with an average age of 17.88. The hospital professionals had a mean age of 39.27 years, and 9.54 mean years of experience in mental health. Ten parents of students and six teachers of students participated, aged, on average 50.1 and 56.16 years, respectively. Teachers had 32.16 years of teaching experience.

The themes representing the participants’ experiences were extracted from the non-participant observations, FGs, informal interviews, diaries and personal letters. Five themes emerged from the material analyzed: a) learning to live together, b) the perception of the illness and of the mentally ill, c) change, d) a test and an opportunity, e) discharge and readmission.

We included the narratives taken directly from the data collection regarding the five themes identified in this study.

### Theme 1. Learning to live together

The patients described how they had to learn to live together and socialize with “healthy” people. Furthermore, the cohousing experience meant having to overcome obstacles such as leaving the hospital, working in a group, and being evaluated by the other participants: “At the beginning everything was difficult, then, little by little I realized that I could do it,… slower, with more fear, but I could, I never imagined that I could do it on my own” (Informal patient interview). On many occasions, patients described that one of the main barriers for interacting with others was the fear of rejection, or being treated as inferior: “I was afraid that they would look down at me, or that they would realize that I was clumsier or that I had difficulty doing things, or that I needed pills to remain calm, or that they wouldn’t want to look at me… because I am like an ill person.” (Patient focus group.)

Both groups described participating together during all activities (games, cooking, cleaning), first formally (programmed groups) and later informally (during spare time). Key aspects were the feeling of camaraderie and togetherness: “In the end we went together everywhere, besides the organized activities, we had a coffee, in our spare time, it was great to have that amount of understanding with them” (Student focus group). The patients reported that nobody treated them differently or talked to them as if they were inferior: “We were together throughout the co-living experience, in groups, I never noticed any foul words, or gestures… I never felt inferior or pointed at. The girls [students] were really good with us” (Patients focus group).

Another common point raised was that both groups were able to learn from one another. During the cohousing initiative, the students and patients shared time, space and performed activities together that favored the exchange of experiences. Many of the conversations were focused on how they were living, what they did before being hospitalized or before participating in the study, or regarding their past and their family. Together with this exchange, the psychologist performed sessions with the patients and the students, both separately and together, in order to facilitate the adaptation, emotional control and management of conflicts. As a result, they learnt together. On the one hand, most students acknowledged that taking decisions (using drugs) always had consequences and, on the other hand, all the patients described how, on many occasions, they had made mistakes, regarding their behavior with their family and their drug addiction: “I am afraid of making decisions, but I am even more afraid of freezing and doing nothing.” (Letter by a student), “They have seen people who were like them and who have taken the wrong steps, ending up how they are now.” (Parent focus group). Furthermore, they learnt to evaluate their capabilities: “With them, everything is out in the open, you can’t fool yourself, you are what you are, and if you are not prepared, this becomes visible during the cohousing experience, and if you cannot manage a few days, how are you going to live outside on your own?” (Patient focus group)

### Theme 2. The perception of the illness and of the mentally ill

The students described a change in the way they view those who are ill. Before they branded them as being “crazy”, aggressive, doing odd things, trying to grab attention on the street, walking strangely, people who acted clumsier while doing things and while speaking: "… they have transformed from being ill to being normal people.” (Student focus group). The final consequence of this was that they were scared and avoided contact with those affected: “They took care of us much more then we took care of them. They had no problem from the beginning, [they didn’t experience] the rejection that perhaps we have since the beginning.” (Student focus group). Afterwards, the ill individuals were described as: “very good people”, and “not inferior”, or, in the words of one of the students: “they can have difficulties but that doesn’t mean that they do things wrong”. Even the parents described how their daughters had modified their perspective: “When my daughter sees someone who is ill, instead of saying ‘that person is crazy or a madman’, she now says that that the person is ill, in need of treatment and that the person is going to be able to live a normal life like the rest of people.” (Parent focus group). Furthermore, students remarked that mental illness has nothing to do with malice or being bad people: “If those who are ill get aggressive it’s because you have done something to them. It’s unusual for them to do or say something to you for no reason. Also, not just because you are mentally ill are you going to be bad. There are many normal people who are much worse.” (Student focus group).

During the different stages of data collection, the professionals and parents described the existence of a very fine frontier between being healthy and being ill, and that this can happen to anyone: “Until it happens to you, you aren’t aware of how easy and how common it is to have a mental illness.” (Professional focus group). It is not something that can be controlled or avoided: “Any day you can have a bad situation and then, suddenly, bam! They diagnose you and you are now mentally ill.” (Teacher focus group). Participants acknowledged having difficulties trying to recognize who was ill just at a glance: “On the streets, in normal life you cannot distinguish who is normal and who has a mental illness.” (Student focus group).

Students mentioned how, in society, contact with those who are ill is avoided, it is generally thought that mental illness “is contagious”. It is said that if you are in contact with ill people, something rubs off, therefore, the solution is to keep a distance from them: “The first thing they told me when they knew I was going to the cohousing project was: ‘let’s see if you come out a bit affected’. I don’t think they really mean it, but that’s what they say to you.” (Student focus group). The society does not provide a real image of patients, they project a distorted and grotesque image: they are always the bad ones, the crazy ones, bringing up thoughts of asylums, or as people who may harm you, etc. This is what is projected in films, in the news etc., however, it is not a reflection of real life: “People, society, they don’t know what they are really like, they have no idea. They just say, “they are mad”, full of complexes… In the end, people talk without knowing.” (Student focus group).

### Theme 3. Change

Students remarked that a change and a transformation had taken place within them. They felt more self-confident but, at the same time, they had many doubts, questioning everything about their life: “It’s like someone has removed a bandage from in front of my eyes” (Student focus group), “It was like having lived in a bubble. What I thought was life was not truly real…” (Student focus group). The contact with patients has made them reflect on many dimensions of their life: “It has been like an existential stir.” (Teacher focus group), “You couldn’t stop thinking that this was really harsh, everything they have lived through and suffered from. How can I say that my life is difficult? There came a time when I didn’t even know who I was or what I really wanted.” (Student focus group). Furthermore, for these students, living with patients was like seeing themselves in a mirror. Some described how, at times, they felt ridiculous for leading a life with commodities, considering that they had been wasting their life: “I realized that I didn’t recognize myself, as if I were mistaken all this time.” (Student focus group), “You hear so much and you see that you have your whole life ahead of you yet, you waste your time with silly things…” (Student focus group).

The parents and teachers described perceiving students as more mature, by discussing their positions, making decisions, and reflecting upon their motivations: “We were talking about going to university, choosing a career and she said she was scared of taking steps forward, but she was even more scared of not taking them”. (Parent focus group). Some parents were surprised by this acquired maturity and the harshness of this new perspective: “My daughter told me… The world is no longer all peaches and cream as we thought, but that does not mean that it is bad”. (Parents focus group). For both the parents and the teachers, the cohousing experience impacted the students to such a great extent because it made them undergo very emotionally intense experiences: “One week after the cohousing experience my daughter came and told me how one of the patients explained to her the correct way to commit suicide, how the arm should be cut longitudinally, not perpendicularly, so that that way they wouldn’t be able to stich and repair the artery… She is 17 years old, she shouldn’t know this…”(Parents focus group), (I spoke to X [name of patient] and she told me that each time she tried to kill herself she learnt a bit more. She was an expert in trying to die and each time she got better at it… she told me all the ways to do it) (Student focus group). This intensity is due to the “real” nature of the experience: “… it’s not a lesson from a book, they have lived with mentally-ill people, they have seen the consequence of making decisions first-hand. The key has been the experience, what they have gone through is etched in their memories forever.” (Teacher focus group).

### Theme 4. A test and an opportunity

The cohousing experience was perceived as both a test and an opportunity for patients. Thanks to this experience, it was possible to test the patients’ capabilities under controlled conditions while at the same time overcoming barriers.

The patients perceived the cohousing experience as an opportunity for demonstrating their ability to live with healthy people, work in a team, and refrain from substance abuse: “It’s an opportunity that they have given us and I don’t plan to waste it, I shall demonstrate to both them and myself that I can do it” (Informal patient interview). It was a way of demonstrating that they are prepared for discharge. Also, it was perceived as a test to be able to live with others outside the hospital, and discover their limits. In this test, there were risks of failure, and uncertainties, because not everything is controlled (i.e. facing the students’ opinions of themselves, and facing possible rejection): “It’s an opportunity but at the same time a test. A test that you can be successful with or mess up… I was very unsure regarding what would happen… would I be able to do it? (Patient focus group). On the other hand, this enabled them to test themselves under controlled situations, before the “official discharge”. In this manner, if they did not succeed, they knew they could return to hospital, without considering this a readmission or a relapse: “It is like a rehearsal, where you can see for yourself what it can be like to not live in the hospital… it gives you an idea of what it will be like outside, and on top of it all you feel anxious as you know that if you fail, and you get put in again, what will they say about you, the looks and comments you will get from other people.” Patient focus group).

### Theme 5. Discharge and readmission

Cohousing represents a step closer towards being discharged. However, a flip side to discharge exists. On the one hand, it can signify recovering “the lost freedom”, abandoning the norms and the hospital control: “This is like a jail, only worse. In jail, you know when you get in and when you are getting out, not here…” (Patient focus group). However, at the same time, many patients avoid leaving the hospital, as they are afraid of rejection, using substances again, or that the daily responsibilities may go beyond their capabilities: “…I have fought for discharge, and I wanted it, but then I have gotten out and realized that I no longer want it. Nobody helps me, living outside [the hospital] is very complicated, it’s expensive, you don’t have the benefits that you may have at the hospital…” (Patient focus group).

During the cohousing experience, one of the patients suffered a crisis and had to be taken to the hospital and abandon the cohousing program. As a result, patients began to narrate their own past experiences of being admitted to hospital and suffering relapses. Thus, for a patient who has been discharged, readmission due to a relapse is experienced as a failure, as it demonstrates the patient’s inability to live by their own means. Furthermore, together with the feelings of failure, there is a feeling of blame: “You cannot avoid feeling guilty, nobody makes you leave, you do it and you want to get out but you don’t realize that you are unprepared… Guilty for not having foreseen it.” (Patient focus group). Patients acknowledged that, in previous readmissions, they felt they had not done all that was in their hands to remain discharged from hospital, i.e. to avoid relapsing and avoiding substance abuse: “I messed up, after all my efforts, I lost an opportunity, I have done things wrong.” (Patient focus group).

## Discussion

The most relevant results of this study include: students and patients participating and learning together; a slim barrier between health and disease, a transformation taking place in students, regarding their self-perception, patients testing their ability to live outside the hospital, and discharge, experienced as both a liberation and a difficulty, whereas relapse and readmission are experienced as a failure.

Our results show how, during the cohousing experience, patients and students relate with each other, learning and overcoming barriers together. A number of programs have been developed to promote the contact between adolescents and patients in order to break the stigma, improve the understanding of the mentally ill, promote mental health among the young and prevent substance abuse [30–32]. Previous studies [30,31] have suggested that contact-based education on mental illnesses may be an important component of diversity training for adolescents, ideally before stereotypes of mental illness begin to become established. In circumstances of equal status and common goals, greater contact with members of a stigmatized group (i.e. schizophrenia patients) may exert a generic effect, by replacing faulty perceptions and reducing prejudice and discrimination [30–32]. Conrad et al. [61], Schulze et al. [62], and Thornicroft et al. [63] showed that, in the case of certain target groups, such as students, social-contact-based interventions against stigma usually achieve short-term attitudinal improvements (this is less clear in the long-term), and, although less frequently, can also lead to an increase in knowledge of the same.

Our results reveal a change in the female students’ vision of the patient and mental illness. However, with the design used in this study, we cannot describe how this change of perspective has occurred, nor whether this change will be long-lasting. However, these findings do seem to be relevant for participants who have taken part in the experience. The students also perceived that the overall society continues to reject mental illness. In Spain, Muñoz et al. [64] reported that an abusive and incorrect use of terms related with schizophrenia exists in the press, radio and television. The coverage is scarce and the news analyzed often contains stigmatizing contents that highlight the negative stereotype. This inappropriate treatment on a social level may be due to the fact that the meaning of the term “schizophrenia” is based on a biological and deterministic vision of the illness, which has influenced the ways in which people with the diagnosis may find themselves perceived in their interactions with professionals, family and the wider society, and therefore influence how they may come to see themselves [65].

Our results reveal that the experience of patients living together with students has provided an opportunity to overcome barriers, helping patients to demonstrate their ability to live outside the hospital, relate with healthy people, share, work on common goals, and establish friendships. Anderson [40] described how schizophrenia patients know that they are mentally ill and that they make a special effort to test reality by asserting their autonomy. Furthermore, they see reality as providing an opportunity to analyze themselves. Recovery-oriented practice focuses on the recovery of such factors as hope, empowerment, meaning, identity, and connectedness [10]. Also, previous studies [66,67] reported that mentally ill people understand recovery as becoming more capable and more autonomous in activities that allow them to integrate into the community, such as having a job. On the other hand, prior studies show that schizophrenia patients, by keeping occupied and working with other people, develop the ability to interact, and improve their social relations, [67,68] as well as building friendships [69]. Previous studies [70,71] have pointed out how these interventions must be established in order to help people who suffer from persistent and severe mental health problems that limit their daily life and their ability to fulfil their personal objectives, i.e. these interventions must be directed towards the development of strategies to face the difficulties of daily living. This will strengthen the individuals’ decision making and skill development to enable them to live in their environment, establish relations with others, and avoid the use of substances.

Our results showed that patients feel that their discharge is both a source of liberation as well as a risk. The fear of failure, of having a relapse, and facing readmission is very real. At the SJPH, before hospital discharge, patients receive home support, living with other patients in the same apartment under regular supervision. However, there is no intermediate step between being at hospital and receiving supported housing. At the time of the study, patients diagnosed with schizophrenia at the SJPH (n = 200) presented between 32–36 hospitalizations per year, remaining in the community for 33 months, on average, without the need for being readmitted. Previous studies [72,73] have highlighted the difficulty of managing life following discharge, together with feelings of loneliness. Patients want to return to their life prior to hospitalization, however, they become frustrated as they are unable to make the appropriate decisions to achieve this. Previous studies [42,73] have shown that relapses may represent a danger that will accompany the patient for the rest of their life outside the hospital, making it difficult to make plans for the future, as individuals with schizophrenia must always remain vigilant. Nordick & van Heugten [42] describe recovery as a process with three stages, from chaos, to dynamic contemplation, and leading to eventual wise integrative adaptation. In this study, the impetus toward acceptance of the risk of relapse, and adaptation was provided by dangerous events (substance abuse, and avoiding medication) that forced participants to perceive the risks of ignoring the impact of the illness on their lives. The experience of relapse is highly individual and risk factors exist, as well as protectors. Sariah et al. [74] described personal and environmental factors, both with risks and protective factors. Within the risk factors, these authors describe: the lack of adherence to medication and taking drugs (personal), the absence of family support and stressful events (environmental) [74]. On the other hand, the protective factors are: adherence to medication, employment, religion (personal), family support, support from other patients, visits and professional follow-up, and a therapeutic relationship with other professionals [74]. There are differences between the personal perception of the recovery of the patients themselves and the clinical recovery (decrease of symptoms) expected by professionals, which may condition the expected treatment results [73, 75]. Therefore, having a job, having support from the family and the surroundings, performing activities that are meaningful for the patient and going to therapy, help improve the patient’s feeling of wellbeing [76].

It is important to note that, in case studies, different units of analysis are included which enable us to understand the phenomenon in greater depth, as well as serving as a triangulation technique to control the quality of the study and provide information from another perspective that is different to that of the patients [47,56,57].

### Limitations

In the first place, during the study we were unable to include participants from the patients’ family environment, as they declined to participate in the study. Indeed, prior studies [73,77] have shown how the family can experience situations of emotional exhaustion. Second, the study included a small number of participants and the cohousing experience was limited to four days. Third, this study has not quantified the efficacy of the cohousing experience. The qualitative nature of this study meant that the focus was on describing the experiences of the participants and, therefore, these findings cannot be generalized. Also, we are lacking longitudinal data or a comparison group. Finally, there were differences regarding the age and gender of the participants who participated in the cohousing experience. In order to control for this difference, within the cohousing experience, mixed groups were formed haphazardly, to avoid grouping by age or sex.

## Conclusions

Our findings can help us to better understand the relationships and perceptions between people with mental illness and those without. There are different forms of supported housing such as Housing First [78], Permanent Supportive Housing [79,80], and Recovery Housing [81]. All these modalities share the fact that they provide a safe, stable and permanent place to enable the person to feel safe, helping people to develop their capacities, have a more independent life, and feel better integrated with their local community [81]. All these modalities are based on cohousing between patients in a collective or individual manner, supported and supervised by professionals [80,81]. Our results highlight another alternative for recovery housing research: the development of cohousing projects for patients with healthy people over short time periods. Cohousing experiences and direct exchanges between psychiatric patients and adolescents, may be a therapeutic alternative for promoting mental health, preventing mental illness, reducing addiction to substances, and, even, discrimination, while increasing confidence and helping individuals with schizophrenia in their decision making. These results may be used to develop cohousing programs in controlled environments. These experiences can also include other types of residents (co-residents) such as healthcare and social workers, even family members of the patients themselves.

## Supporting information

S1 File “Meet the Hospital” program. (DOC)

S2 File Permission to use photos of Respaldiza House. Hospitaller Order of Saint John of God.
(PDF)

S3 File Procedure for the cohousing initiative. (DOC)

S4 File Focus group: Question guide for patients with schizophrenia. English version.
(DOC)

S5 File Focus group: Question guide for patients with schizophrenia. Spanish version.
(DOC)

S6 File Focus group: Question guide for participants. English version.
(DOC)

S7 File Focus group: Question guide for participants. Spanish version.
(DOC)

S8 File Clinical Research Ethics Committee approval—University Rey Juan Carlos. (PDF)

S9 File Clinical Research Ethics Committee approval—Hospitaller Order of Saint John of God Foundation. (PDF)

S10 File Permission from the medical direction at Hermanos San Juan de Dios Psychiatry Hospital. (PDF)

S11 File Ethical considerations for students and schizophrenia patients. (DOC)

S1 Photo Respaldiza House photos. (PDF)

S1 Fig Case-study components. (DOCX)
