# Supplementary material for: The impact of a short-term cohousing initiative among schizophrenia patients, high school students, and their social context: A qualitative case study
Source: PLoS One. 2018 Jan 11;13(1):e0190895. doi: 10.1371/journal.pone.0190895 (PMC5764336; doi:10.1371/journal.pone.0190895)
Supplement: S1 File — (DOC) [file pone.0190895.s001.doc]

**“MEET THE HOSPITAL” PROGRAM**

**
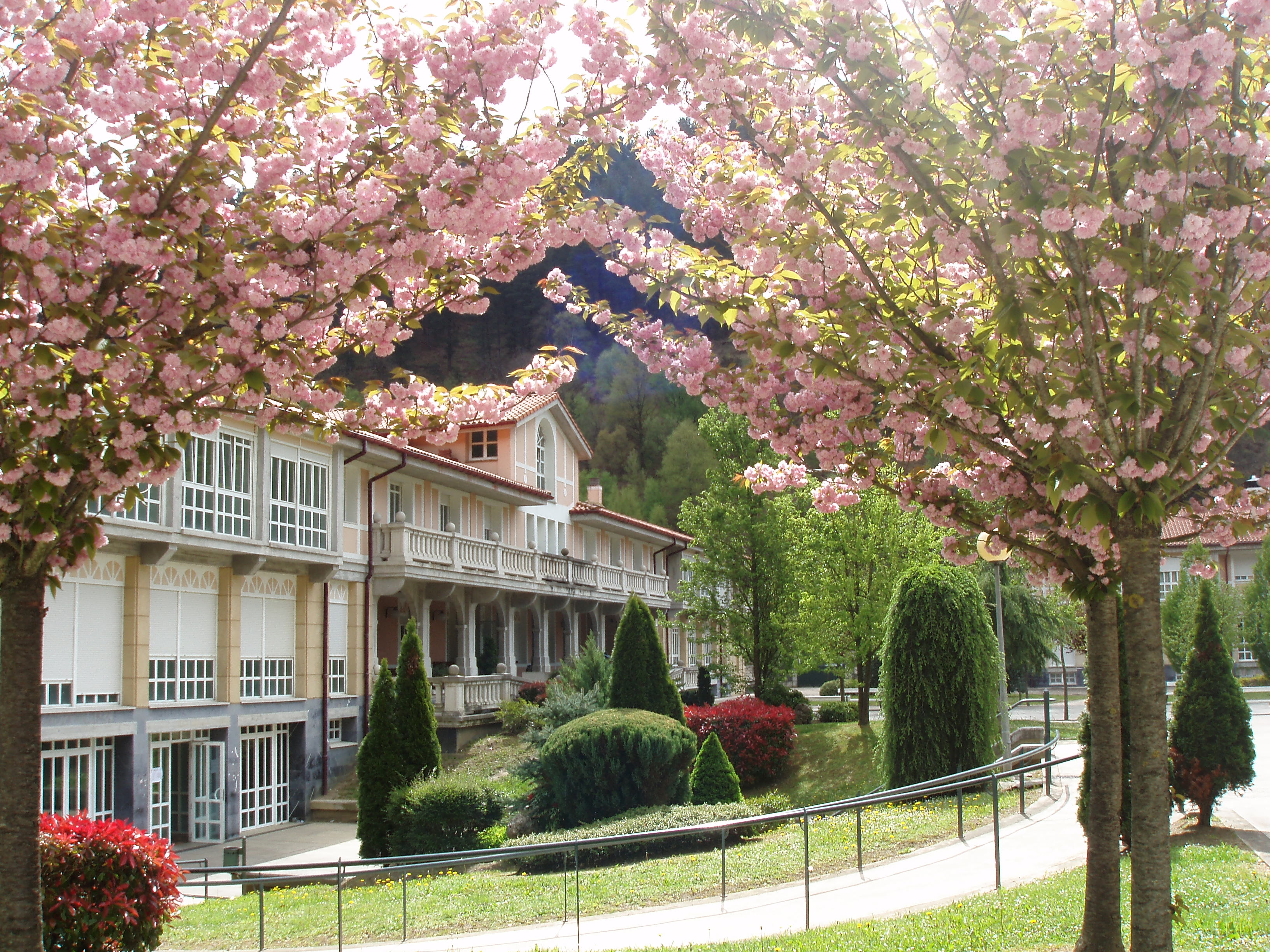
**

**REHABILITATION SERVICE**

**San Juan de Dios Psychiatric Hospital (Arrasate)**

**“MEET THE HOSPITAL” PROGRAM SUMMARY**

This program attempts to fulfill several objectives.

- **Give visibility to mental illnesses, and try to reduce, mitigate and/or eliminate the effect of associated stigmatization.**
- **Enable students to gain first-hand knowledge regarding mental illness.**
- **Show students the true impact of substance abuse.**

Ten years ago, the hospital has observed the effects of drug abuse on mental health. The number of people who are admitted to the hospital under the age of 25, with a clinical diagnosis of a psychiatric pathology, and with the symptoms of substance abuse (dual processes) has increased.

The result of this is that the hospital now undertakes tasks for the prevention of substance abuse. The program seeks to offer **real, objective, up-to-date and proven** information for high school students in an attractive and understandable format, focusing on the use of substances, concretely, cannabis. This is how the **“Meet the hospital”** program was born.

At the end of the year, the number of students that participate in this new program ranges between 400 – 450.

**CONTENTS OF THE “MEET THE HOSPITAL” PROGRAM**

The program consists of several activities: a) a first visit to the hospital, b) seminar, and c) sharing experiences.

**a) FIRST VISIT TO THE HOSPITAL**

The visit lasts three hours. The following contents are presented:

**A presentation on the institution and hospital.**

**A talk on the effects of substance abuse**

- Offering a description of drugs that are taken, their components, the interactions these produce at the level of the brain, and the psychopathological disorders associated with abuse.

**Analysis of a real case featuring an adolescent**

- Presentation of the case; assessment of the personal, social, family, clinic, educational, judicial, and economic effects. Discussion on the severity of the case, the responsibility of the patient and family. Proposing ways to help.

**Discussion between the group of students and patients**

- The aim is to gain first-hand knowledge of the experience of a patient in the context of substance abuse.
- This discussion fulfills a double purpose. Students directly learn the consequences of drug abuse, and patients know that their experience may be useful for avoiding substance abuse.

**b) SEMINAR.**

The seminar lasts 3 hours.

This activity is performed in an educational center, and involves the intervention of parents, teachers, and students, while incorporating a group of patients.

Three groups of 10 people are conformed, comprising two teachers from the center, three students, three parents and two patients.

**Methodology**

The three groups work separately on two case studies featuring adolescents. A text or an audio is used to inform the group of the case and evolution of the same.

- Case presentation; assessment of the personal, social, familiar, clinical, educational, judicial and economic consequences involved. Discussion on the severity of the case, the responsibility of the patient, and family and proposing ways to help.

After analyzing both cases, all three groups share their conclusions.

This type of group dynamic enables us to find a space for reflection, where all participants can contribute something.

**c) SHARING EXPERIENCES**

This is an initiative between the San Juan de Dios de Arrasate Hospital, and the teaching institutions that wish to perform experiences aimed at disseminating the reality of mental illness and fighting against the stigma.

- Cohousing program with patients and students.
