# Supplementary material for: The impact of a short-term cohousing initiative among schizophrenia patients, high school students, and their social context: A qualitative case study
Source: PLoS One. 2018 Jan 11;13(1):e0190895. doi: 10.1371/journal.pone.0190895 (PMC5764336; doi:10.1371/journal.pone.0190895)
Supplement: S2 File — Hospitaller Order of Saint John of God. (PDF) [file pone.0190895.s002.pdf]

AUTORIZACIÓN PARA LA PUBLICACIÓN DE FOTOS DE LA FINCA RESPALDIZA

EL **HNO. MIGUEL ÁNGEL VARONA ALONSO** en calidad de Presidente del Patronato de la Fundación San José y San Buenaventura y Superior Provincial de la Provincia de Castilla de la Orden Hospitalaria de San Juan de Dios,

De cara a la publicación de resultados del proyecto de investigación "Perspectiva del estigma sobre personas diagnosticadas de trastorno mental grave" aprobado por la Comisión de Investigación de la Fundación San Juan de Dios y llevado a cabo en el marco de la Convivencia celebrada en 2015 en la Finca que la Provincia de Castilla de la Orden Hospitalaria de San Juan de Dios posee en Respaldiza, en la que durante cuatro días participaron pacientes con enfermedad mental grave del Hospital de Arrasate y alumnas de una Ikastola de Mondragón,

AUTORIZA

la divulgación de fotos de dicha finca y de la casa donde se realizó la convivencia, **siempre que no aparezcan en ellas personas que puedan ser identificadas.**

Por ello firma la presente autorización en Madrid a 3 de noviembre de 2016

Hno. Miguel Ángel Varona Alonso

Presidente del Patronato Fundación San José y San Buenaventura

Superior Provincial. Provincia de Castilla
