# Supplementary material for: The impact of a short-term cohousing initiative among schizophrenia patients, high school students, and their social context: A qualitative case study
Source: PLoS One. 2018 Jan 11;13(1):e0190895. doi: 10.1371/journal.pone.0190895 (PMC5764336; doi:10.1371/journal.pone.0190895)
Supplement: S3 File — (DOC) [file pone.0190895.s003.doc]

**S3 File.** The Cohousing Initiative procedure.

The cohousing initiative and all the activities that took place were directed and supervised by a psychologist and two occupational therapists.

The purposes of the cohousing initiative were: a) to bring the society closer to people with mental illness and with severe mental disorders, b) to devise actions against the creation of stigmas in a young generation, c) to enable patients to assess whether they have sufficient resources to face the responsibilities and commitments necessary to live in a normalized context, d) to evaluate the learning performed at the Rehabilitation Service in a real context, and the openness of the Hospital towards its immediate environment, e) to have students live with patients with a history of substance abuse and therefore enable students to observe first-hand the effects of the drugs on those affected.

The patients slept on the first floor and the students slept on the second floor, together with the psychologist and the two occupational therapists.

Prior to beginning the cohousing experiment with the patients, the rehabilitation service worked on a series of skills for everyday life, as well as the patient’s social skills, teaching healthy habits, and improving their awareness of the illness and adherence to treatment. In this manner, during the cohousing period, the groups were then divided by activities, in which patients and students were made to work and interact with each other. Thus, the following groups were established: the medication group, meals group, social skills group, group for leisure and free time, psychoeducational group and the group for cognitive stimulation.

The tasks for each group were:

- Medication group: in the morning, before breakfast, each group had to organize their medication for the whole day.
- Meals group: each group was in charge of breakfast, lunch and dinner for one day. This same group was in charge of dishwashing at the end of each meal and then leaving the dishes prepared for later use.
- Social skills group: every day a group worked on a subject that had to do with social skills, relational kills and interpersonal communication. Concrete cases were analyzed in order to later share what each group has worked on.
- Cognitive stimulation group: all the groups performed a “cognitive gymkhana” activity, in which they had to resolve a series of exercises involving logic, text analysis, calculations, cryptography, with the aim of resolving problems and learning how to work in a team.
- Psychoeducational: this group worked on themes related to healthy habits and awareness of the illness.
- Leisure and free time: Every afternoon, each group left the guest house to visit close by touristic sites, strengthening the mutual knowledge between patients and students and working on personal resources.
