# Supplementary material for: The impact of a short-term cohousing initiative among schizophrenia patients, high school students, and their social context: A qualitative case study
Source: PLoS One. 2018 Jan 11;13(1):e0190895. doi: 10.1371/journal.pone.0190895 (PMC5764336; doi:10.1371/journal.pone.0190895)
Supplement: S4 File — English version. (DOC) [file pone.0190895.s004.doc]

**S4 File. Focus Group: Question guide for patients with schizophrenia. English version.**

| Research topics | Questions asked |
| --- | --- |
| Cohousing | How has your experience with this cohousing program been? What do you consider has been the most relevant aspect of this experience?  What prior ideas did you have of living with people WITHOUT a mental illness? What do you think people have thought about you?  Have you encountered any facilitator or obstacle during the cohousing experience? |
| Mental illness | What do you think about mental illness? How has your experience been? |
| People with mental illness | What do you think about people diagnosed with a mental illness? How is your relationship with other mentally ill people? |
| Family | What is your relationship with your parents, children and/or the people surrounding you?  What do you consider is the most important/relevant thing for you concerning these relationships? |
| Social context | What is the most relevant/important thing for you in your relationships with people?  From your point of view, how do you think people perceive mental illness, people with mental illnesses and the families of people with mental illness? |
