# Supplementary material for: The impact of a short-term cohousing initiative among schizophrenia patients, high school students, and their social context: A qualitative case study
Source: PLoS One. 2018 Jan 11;13(1):e0190895. doi: 10.1371/journal.pone.0190895 (PMC5764336; doi:10.1371/journal.pone.0190895)
Supplement: S5 File — Spanish version. (DOC) [file pone.0190895.s005.doc]

**S5 File.** Grupo Focal: Guía de preguntas para pacientes con esquizofrenia. Spanish version.

| Áreas de investigación | Preguntas |
| --- | --- |
| Convivencia | ¿Cómo ha sido su experiencia con esta convivencia? ¿Qué ha sido para usted lo más relevante de esta experiencia?  ¿Qué ideas previas tenías de la convivencia con personas SIN enfermedad mental? ¿Qué crees que ha pensado la gente de ti?  ¿Has encontrado algún facilitador u obstáculo en la convivencia? |
| Enfermedad mental | ¿Qué piensa sobre la enfermedad mental? ¿Cómo es tú experiencia? |
| Persona con enfermedad mental | ¿Qué piensa sobre las personas diagnosticadas de una enfermedad mental? ¿Cómo es su relación con otros enfermos? |
| Familia | ¿Cómo es tu relación con tus padres, hijos/as y/o las personas de tu entorno/alrededor?  ¿Qué es lo más relevante/importante para ti de esas relaciones? |
| Entorno social | ¿Qué es lo más relevante/importante para ti de las relaciones con la gente?  Desde su punto de vista ¿cómo cree que la gente percibe la: enfermedad mental, a las personas con enfermedad mental, y a la familia de personas con enfermedad mental? |
