# Supplementary material for: The impact of a short-term cohousing initiative among schizophrenia patients, high school students, and their social context: A qualitative case study
Source: PLoS One. 2018 Jan 11;13(1):e0190895. doi: 10.1371/journal.pone.0190895 (PMC5764336; doi:10.1371/journal.pone.0190895)
Supplement: S6 File — English version. (DOC) [file pone.0190895.s006.doc]

**S6 File. Focus Group: Question guide for participants. English version.**

| Research topics | Questions asked |
| --- | --- |
| Cohousing | How has your experience with this cohousing program been? What do you consider has been the most relevant aspect of this experience? |
| Mental illness | What are your thoughts regarding mental illness? |
| People with mental illness | What do you think about people diagnosed with a mental illness? What prior ideas/opinions did you have regarding people with mental illness? |
| Family | What do you think about the partners and families of a person with mental illness? |
| Social context | From your point of view, how do you think people perceive mental illness, people with mental illnesses and the families of people with mental illness? |
