# Supplementary material for: The impact of a short-term cohousing initiative among schizophrenia patients, high school students, and their social context: A qualitative case study
Source: PLoS One. 2018 Jan 11;13(1):e0190895. doi: 10.1371/journal.pone.0190895 (PMC5764336; doi:10.1371/journal.pone.0190895)
Supplement: S7 File — Spanish version. (DOC) [file pone.0190895.s007.doc]

**S7 File**. Grupo Focal: Guía de preguntas para participantes. Spanish version.

| Áreas de investigación | Preguntas |
| --- | --- |
| Convivencia | ¿Cómo ha sido su experiencia con esta convivencia?  ¿Qué ha sido para usted lo más relevante de esta experiencia? |
| Enfermedad mental | ¿Qué piensa sobre la enfermedad mental? |
| Personas con enfermedad mental | ¿Qué piensa sobre las personas diagnosticadas de una enfermedad mental? ¿Qué ideas/opiniones previas tenías de las personas con enfermedad mental? |
| Familia | ¿Qué piensa sobre las parejas, familias de una persona con enfermedad mental? |
| Entorno social | Desde su punto de vista como cree que la sociedad/ la gente percibe: la enfermedad mental, las personas con enfermedad mental, y a la familia con algunos de sus miembros con enfermedad mental. |
