# Supplementary material for: The impact of a short-term cohousing initiative among schizophrenia patients, high school students, and their social context: A qualitative case study
Source: PLoS One. 2018 Jan 11;13(1):e0190895. doi: 10.1371/journal.pone.0190895 (PMC5764336; doi:10.1371/journal.pone.0190895)
Supplement: S8 File — (PDF) [file pone.0190895.s008.pdf]

D. José Luis del Barrio Fernández, Secretario del Comité de Ética de la Investigación de la Universidad Rey Juan Carlos,

CERTIFICA

Que este Comité ha evaluado el proyecto de investigación titulado:

PERSPECTIVA DEL ESTIGMA SOBRE PERSONAS DIAGNOSTICADAS DE TRASTORNO MENTAL GRAVE (PROYECTO RESPALDIZA 2015)
Con número de registro interno: 051020154215

y considera que:

• Se cumplen los requisitos éticos necesarios del protocolo en relación con los objetivos del estudio y están justificados los riesgos y molestias previsibles para los participantes.

• La capacidad del investigador y los medios disponibles son apropiados para llevar a cabo el estudio.

Por lo que ha decidido emitir un dictamen **FAVORABLE** para la realización de dicho proyecto, cuyo investigador principal es **Don DOMINGO PALACIOS CEÑA** de la Facultad de Ciencias de la Salud

Lo que firmo en Móstoles a 18 de diciembre de 2015

UNIVERSIDAD REY JUAN CARLOS
MCMXCVI
COMITÉ DE ÉTICA DE LA INVESTIGACIÓN

Firmado: D. José Luis del Barrio Fernández
