# Supplementary material for: The impact of a short-term cohousing initiative among schizophrenia patients, high school students, and their social context: A qualitative case study
Source: PLoS One. 2018 Jan 11;13(1):e0190895. doi: 10.1371/journal.pone.0190895 (PMC5764336; doi:10.1371/journal.pone.0190895)
Supplement: S9 File — (PDF) [file pone.0190895.s009.pdf]

INFORME DE LA COMISIÓN DE INVESTIGACIÓN DE LA FSJD.

D. Gerardo Díaz Quirós, Presidente de la Comisión de Investigación de la Fundación San Juan de Dios.

CERTIFICA:

Que esta Comisión ha evaluado la propuesta del Investigador Principal D. Domingo Palacios Ceña para que se realice el estudio de investigación, código de protocolo, 01/2015, Nº 01 titulado "Perspectiva del estigma sobre personas diagnosticadas de trastorno mental grave (Proyecto Respaldiza 2015)".

Y tomando en consideración las siguientes cuestiones:

- La pertinencia del estudio, teniendo en cuenta el conocimiento disponible, así como los requisitos del Real Decreto 223/2004, de 6 de febrero por el que se regulan los ensayos clínicos con medicamentos o productos sanitarios; o la Ley 14/2007, de 3 de julio, de investigación biomédica, y las normas que lo desarrollan y su realización es pertinente.
- Los requisitos de idoneidad del protocolo en relación con los objetivos del estudio, justificación de los riesgos y molestias previsibles para el sujeto, teniendo en cuenta los beneficios esperados.
- El seguro o la garantía financiera previstos son adecuados/
- El procedimiento para obtener el consentimiento informado, incluyendo la hoja de información para los sujetos y el plan de reclutamiento de sujetos previstos son adecuados, así como las compensaciones previstas para los sujetos por daños que pudieran derivarse de su participación en el ensayo.
- El alcance de las compensaciones económicas previstas y su posible interferencia con el respeto a los postulados éticos.
- La capacidad del investigador y sus colaboradores y las instalaciones y medios disponibles son apropiados para llevar a cabo el estudio.

Esta Comisión emite un

DICTAMEN FAVORABLE para la realización de dicho proyecto en España.

Lo que firmo en Madrid, a 9 de Octubre de 2015

Firmado:

FUNDACIÓN SAN JUAN DE DIOS

Presidente (G. Díaz Quirós)
