# Supplementary material for: The impact of a short-term cohousing initiative among schizophrenia patients, high school students, and their social context: A qualitative case study
Source: PLoS One. 2018 Jan 11;13(1):e0190895. doi: 10.1371/journal.pone.0190895 (PMC5764336; doi:10.1371/journal.pone.0190895)
Supplement: S10 File — (PDF) [file pone.0190895.s010.pdf]

CONFORMIDAD DE LA DIRECCIÓN MÉDICA/TÉCNICA DEL CENTRO

Dª IZASKUN ANTIZAR MORO, como Directora del hospital/centro HOSPITAL SAN JUAN DE DIOS DE ARRASATE

C E R T I F I C A :

Que conoce la propuesta realizada por el IP DOMINGO PALACIOS CEÑA_ para que sea realizado en este hospital/centro el estudio de investigación, CÓDIGO de protocolo, ___________, versión1 de fecha 30 SEPTIEMBRE 2015 titulado

"PERSPECTIVA DEL ESTIGMA SOBRE PERSONAS DIAGNOSTICADAS DE TRASTORNO MENTAL GRAVE (PROYECTO RESPALDIZA 2015)"

y que será realizado por DR. DOMINGO PALACIOS CEÑA, como Investigador Principal.

Que acepta la realización de dicho estudio de investigación en este hospital/centro CONDICIONADO a la aprobación del mismo por la Comisión de Investigación de la Fundación San Juan de Dios, y en su caso del CEIC correspondiente.

Lo que firma en Madrid, a 30 de Septiembre de 2015 .

Fdo.

Hospital Psiquiátrico
SAN JUAN DE DIOS
Ospitale Psikiatrikoa
DIRECCION MEDICA
(Arrasate-Mondragon)

ANEXO. MODELO DE CONFORMIDAD DE LA DIRECCIÓN MÉDICA/TÉCNICA DEL CENTRO.
