# Supplementary material for: The impact of a short-term cohousing initiative among schizophrenia patients, high school students, and their social context: A qualitative case study
Source: PLoS One. 2018 Jan 11;13(1):e0190895. doi: 10.1371/journal.pone.0190895 (PMC5764336; doi:10.1371/journal.pone.0190895)
Supplement: S11 File — (DOC) [file pone.0190895.s011.doc]

**S11 File.** Ethical considerations for students and schizophrenia patients.

In the case of the students participating in this study, it is important to note that in Spain, people are considered of a legal age, and with full legal capacity for deciding to participate voluntarily in biomedical studies at the age of 18 years [43]. Only one student was overage (18 years-old), however, the same conservative procedure was applied with all students: first, by obtaining permission and informed consent for participation in the study from both the students and the parents, at the same time. The consent of students was performed together, in the presence of the parents.

On the other hand, patients diagnosed with a mental illness are considered a vulnerable population, due to the risk of receiving some kind of emotional damage during the study, as the research contains questions that could be considered very intrusive, i.e. when questioning personal aspects (habits, use of substances, etc.) [34]. Therefore, we sought the permission and informed consent of all patients, and the study was supervised by the office of the Ombudsman of the Basque people “Ararteko”. Ararteko is an organism in charge of supervising the public institutions and defending the rights of the citizens before the public organisms http://www.ararteko.net/home.jsp?language=es). In the case of people who are incapacitated and/or have no autonomy, this organism assumes their legal guardianship. Besides, at the time of signing the informed consent, a member of the therapeutic team was present, accompanying the researcher.
