# Supplementary figures and images for: The impact of a short-term cohousing initiative among schizophrenia patients, high school students, and their social context: A qualitative case study
Source: PLoS One. 2018 Jan 11;13(1):e0190895. doi: 10.1371/journal.pone.0190895 (PMC5764336; doi:10.1371/journal.pone.0190895)

**S1 Photo.** Respaldiza House Photos.

Figure A

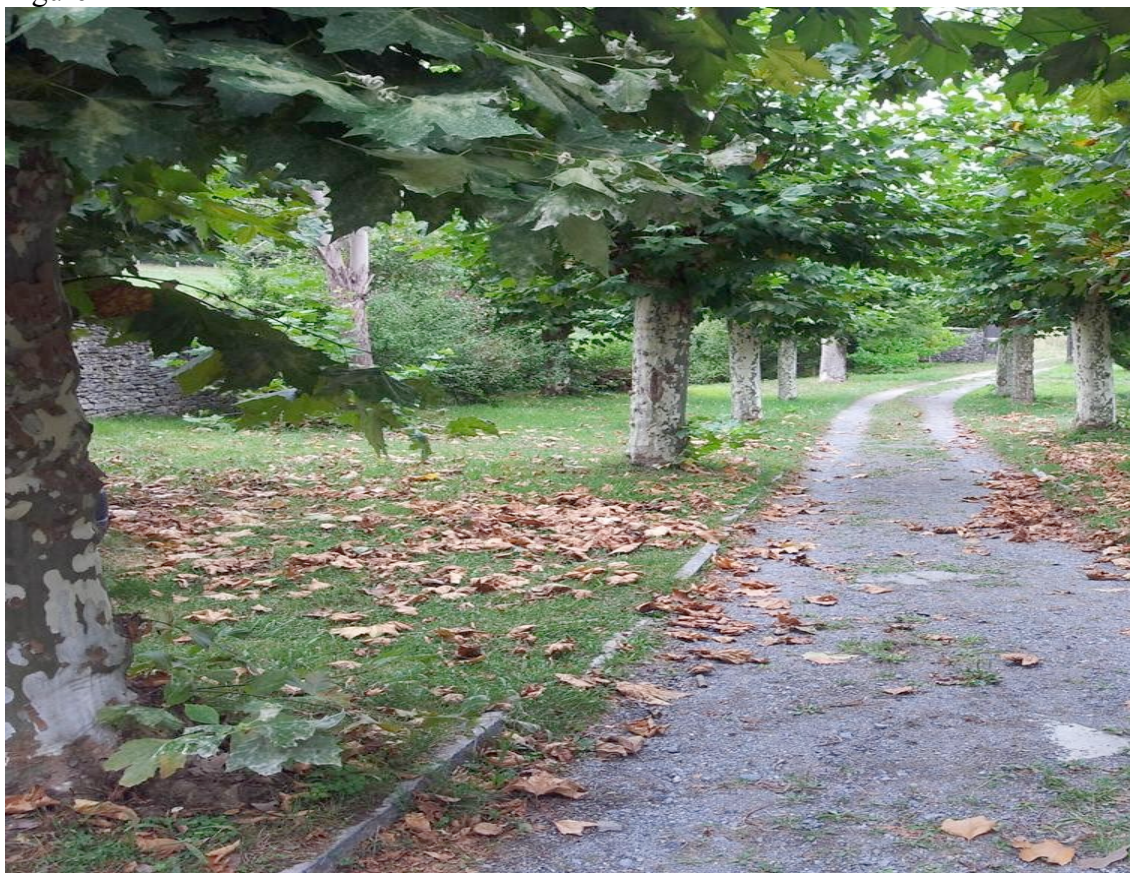

Figure B

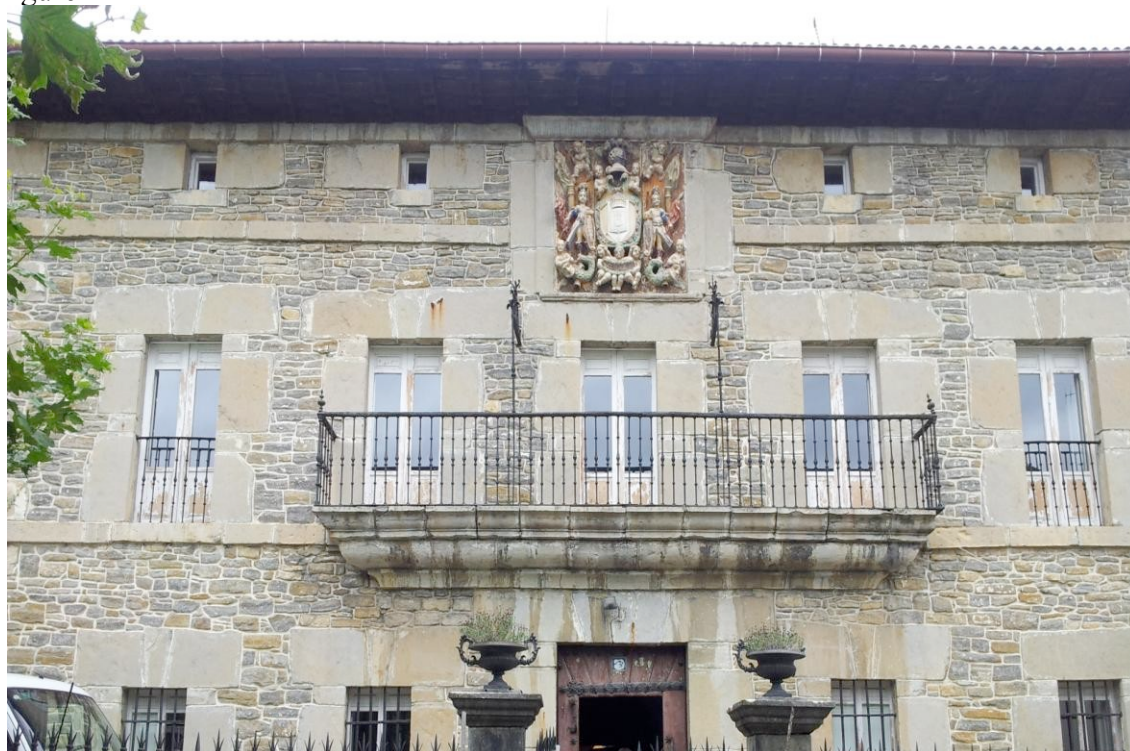

Figure C

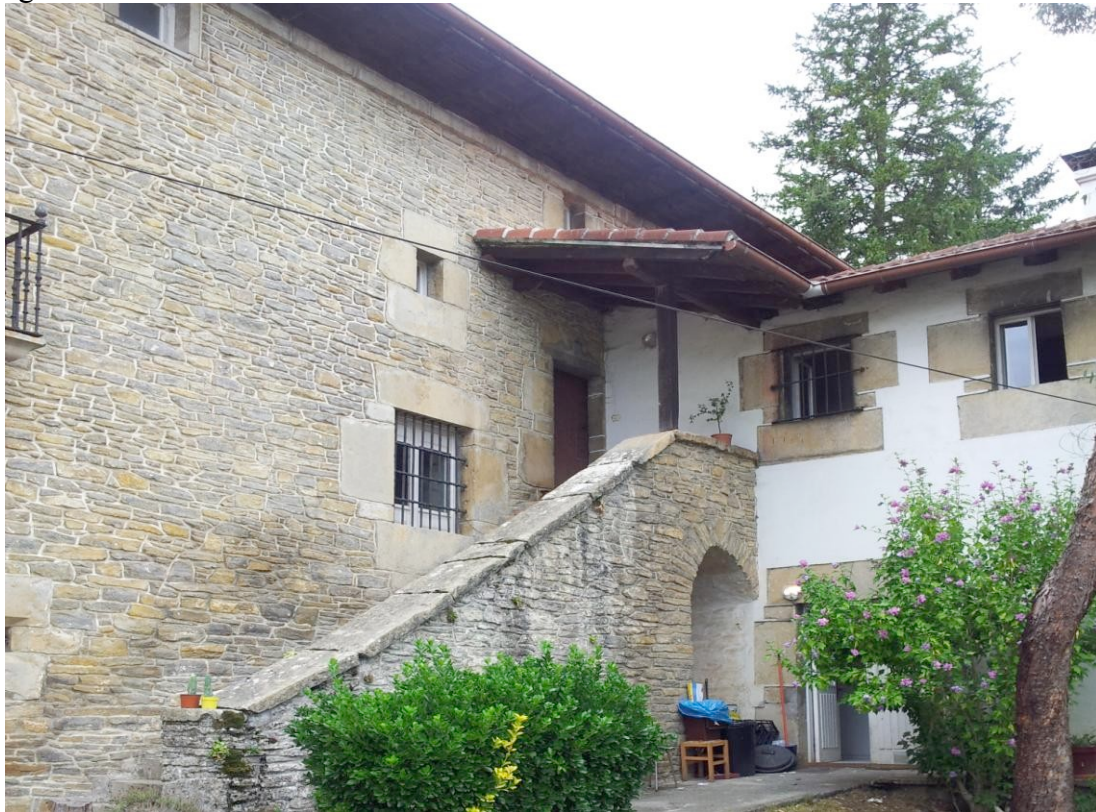

Figure D

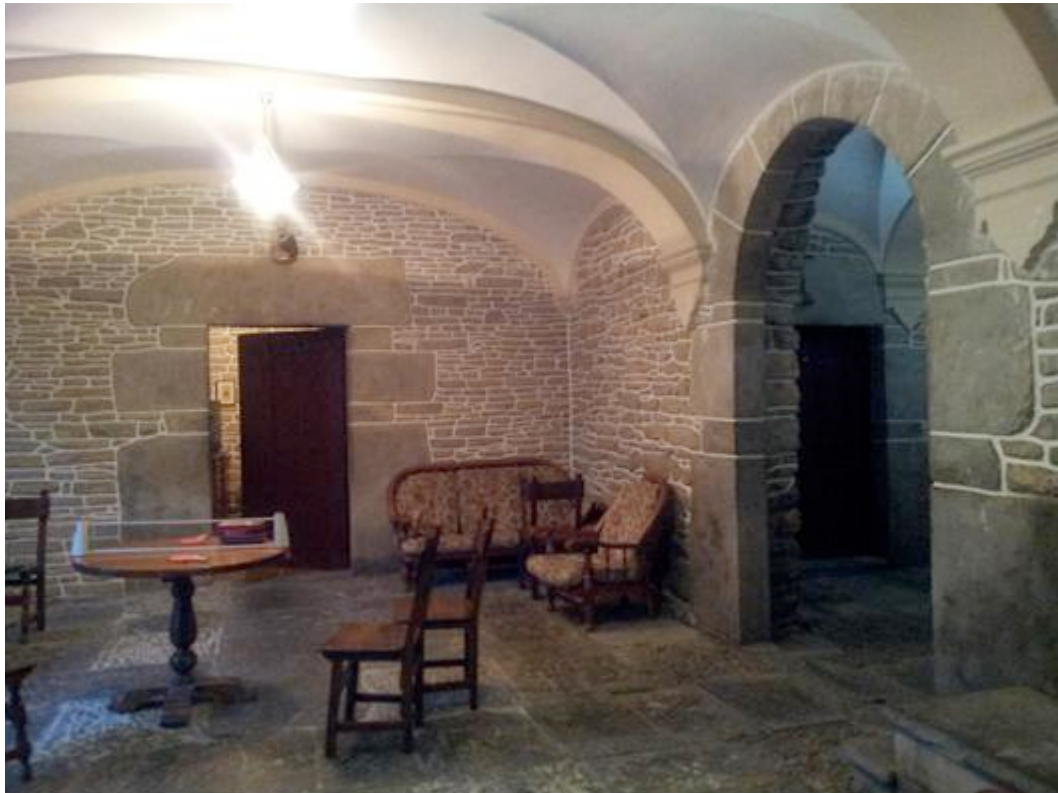

Figure E

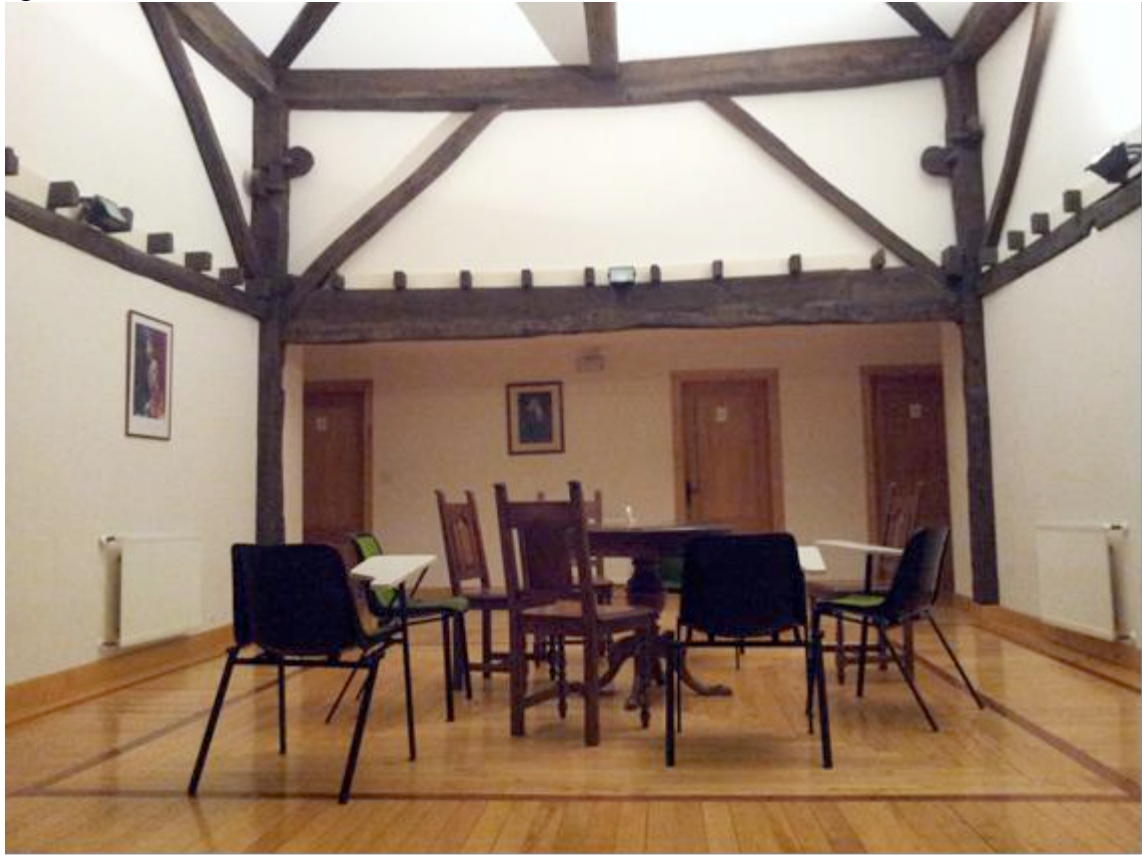

Figure F

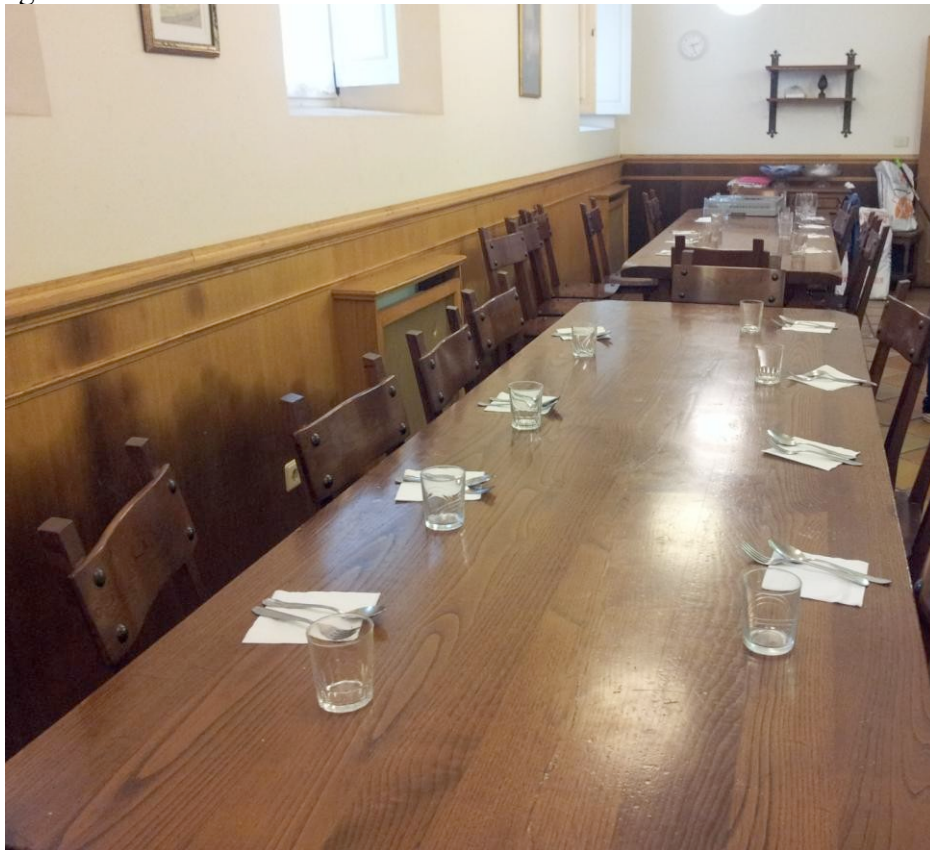

Figure G

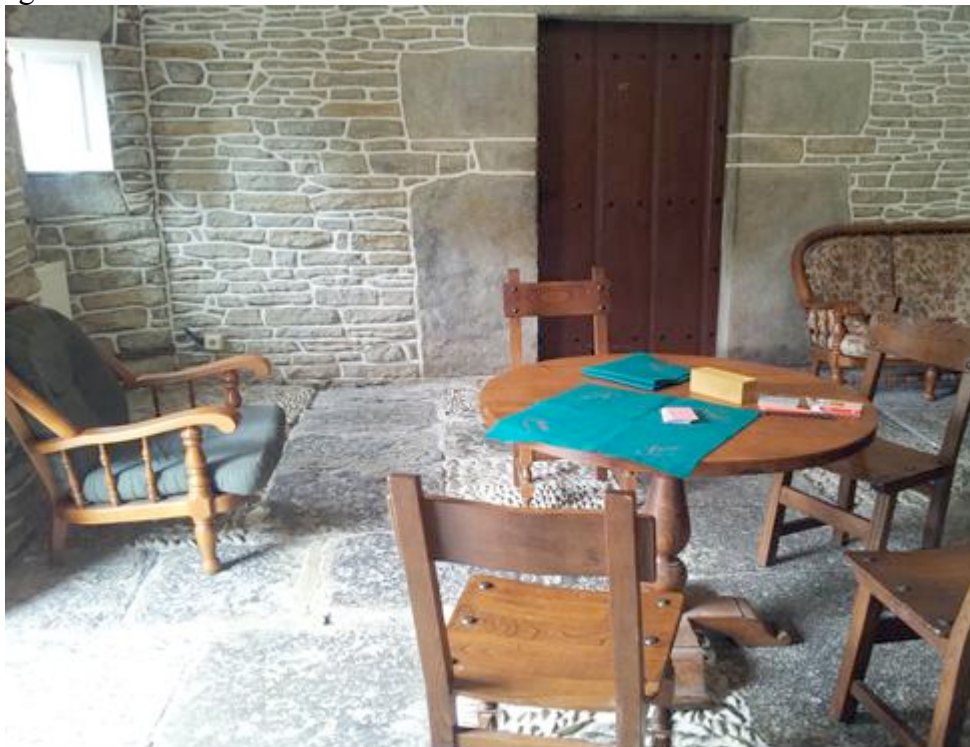

Supplement: S1 Photo — (PDF) [file pone.0190895.s012.pdf]
