# Supplementary material for: The impact of a short-term cohousing initiative among schizophrenia patients, high school students, and their social context: A qualitative case study
Source: PLoS One. 2018 Jan 11;13(1):e0190895. doi: 10.1371/journal.pone.0190895 (PMC5764336; doi:10.1371/journal.pone.0190895)
Supplement: S1 Fig — (DOCX) [file pone.0190895.s013.docx]

**CASE-STUDY**

**Cohousing experience**

**Case-study Unit from Hospital**

Hospital professionals and patients

**Case-study Unit from High-School**

High school teachers and students

**Case-study Unit from Community**

Parents of students and students

Area of Arrasate/Mondragon
